# Exploring the Communal Pathogenesis, Ferroptosis Mechanism, and Potential Therapeutic Targets of Dilated Cardiomyopathy and Hypertrophic Cardiomyopathy *via* a Microarray Data Analysis

**DOI:** 10.3389/fcvm.2022.824756

**Published:** 2022-02-24

**Authors:** Zuoxiang Wang, Qingyue Xia, Wenxing Su, Mingqiang Cao, Yunjuan Sun, Mingyang Zhang, Weixiang Chen, Tingbo Jiang

**Affiliations:** ^1^Department of Cardiology, The First Affiliated Hospital of Soochow University, Suzhou, China; ^2^Department of Medicine, Soochow University, Suzhou, China; ^3^Department of Dermatology, The First Affiliated Hospital of Nanjing Medical University, Nanjing, China; ^4^Department of Plastic and Burn Surgery, The Second Affiliated Hospital of Chengdu Medical College, China National Nuclear Corporation 416 Hospital, Chengdu, China

**Keywords:** bioinformatics analysis, dilated cardiomyopathy, hypertrophic cardiomyopathy, heart failure, ferroptosis, hub genes

## Abstract

**Background:**

Cardiomyopathies are a heterogeneous group of heart diseases that can gradually cause severe heart failure. In particular, dilated cardiomyopathy (DCM) and hypertrophic cardiomyopathy (HCM) are the two main types of cardiomyopathies, yet the independent and communal biological mechanisms of both remain far from elucidated. Meanwhile, ferroptosis is a non-apoptotic form of cell death that has been proven to be associated with cardiomyopathies, but the concrete nature of the interaction remains unclear. Hence, this study explored the pathogenesis and ferroptosis mechanism of HCM and DCM *via* a bioinformatics analysis.

**Methods:**

Six datasets were downloaded from the Gene Expression Omnibus (GEO) database based on the study inclusion/exclusion criteria. After screening the differentially expressed genes (DEGs) and hub genes of HCM and DCM, subsequent analyses, including functional annotation, co-expression, validation, and transcription factors (TF)–mRNA–microRNA (miRNA) regulatory network construction, were performed. In addition, ferroptosis-related DEGs were also identified and verified in HCM and DCM.

**Results:**

We found 171 independent DEGs of HCM mainly enriched in the regulation of ERK1 and ERK2 cascade, while 171 independent DEGs of DCM were significantly involved in cell adhesion. Meanwhile, 32 communal DEGs (26 upregulated genes and 6 downregulated genes) and 3 hub genes [periostin (*POSTN*), insulin-like growth factor-binding protein-5 (*IGFBP5*), and fibromodulin (*FMOD*)] were determined to be shared between HCM and DCM and the functional annotation of these genes highlighted the important position of growth hormone in HCM and DCM. Moreover, we identified activating transcription factor 3 (*ATF3*), lysophosphatidylcholine acyltransferase 3 (*LPCAT3*), and solute carrier family 1 member 5 (*SLC1A5*) as ferroptosis-related genes in HCM and *STAT3* as a ferroptosis-related gene in DCM.

**Conclusion:**

The identified independent and communal DEGs contribute to uncover a potentially distinct and common mechanism of HCM and DCM and ferroptosis-related genes could provide us with a novel direction for exploration. In addition, 3 hub genes could be potential biomarkers or therapeutic targets in patients with cardiomyopathy.

## Introduction

Cardiomyopathies are a heterogeneous group of diseases characterized by structural and functional alterations of the heart, which gradually cause severe heart failure (HF) (1, 2). Dilated cardiomyopathy (DCM) and hypertrophic cardiomyopathy (HCM) are the two most common and prominent types of cardiomyopathies (3). DCM is characterized by ventricular dilatation accompanied by contractile impairment. DCM is one of the main causes of HF, and the prevalence of DCM is 1:250–1:400 cases in the HF population (2, 4). In contrast, the main characteristic of HCM is left ventricular (LV) hypertrophy with primary involvement of the interventricular septum. HCM is a common cause of sudden cardiac death in young athletes, with a prevalence of ~0.2% (5, 6). Recent studies have shown that sarcomere gene mutation is an important factor in the occurrence and development of cardiomyopathy. Although HCM and DCM have some crossover in terms of sarcomere gene mutations, the different types and degrees of sarcomere gene mutations provoke different histopathology and contractile properties, leading in turn to different ventricular morphologies and clinical features (7–9). Although sarcomere biophysics and physiology have provided a new perspective and direction for DCM and HCM research in the past few years, the pathophysiological mechanisms and biological progress of DCM and HCM are still not fully explained (7). The subsequent cascading inflammatory response caused by mutations in the sarcomere gene and the underlying factors independent of the sarcomere remain unclear. Therefore, extensive studies have suggested that the focus of research should be converted from single-gene hypotheses to alternative and complementary mechanisms, and new determinants of cardiomyopathies should be reviewed and screened comprehensively in terms of network medical analysis (10–12). In addition, ferroptosis is a non-apoptotic form of cell death characterized by the iron-dependent accumulation of lipid hydroperoxides to lethal levels (13). Increasingly evidence have shown that ferroptosis plays an essential role in cardiovascular diseases (14–16). Ferroptosis is reported to be associated with many types of cardiomyopathies, yet the exact role and mechanisms of ferroptosis in cardiomyopathy are far from fully elucidated (17). With further study of genotype-to-phenotype correlations in DCM and HCM, the independent pathogenesis between DCM and HCM has gained significant attention (3, 18). Meanwhile, the common therapeutic direction and target of these cardiomyopathies remain to be urgently explored.

The transcription signatures may provide new insights into the pathogenesis of DCM and HCM. Hence, this article sought to identify independent and distinct biological mechanisms of DCM and HCM by bioinformatics analysis and, more significantly, screen the potential common biological pathways of DCM and HCM in order to unearth potential biomarkers and therapeutic directions in these cardiomyopathies. In this article, 4 gene-expression datasets were downloaded from the Gene Expression Omnibus (GEO) for identifying independent and communal differentially expressed genes (DEGs) in HCM and DCM. Then, enrichment analyses of independent and overlapping DEGs were performed in order to uncover the independent and shared biological mechanisms of HCM and DCM. Based on a protein–protein interaction (PPI) network constructed using the search tool for the retrieval of interacting genes (STRING) database and Cytoscape, we performed hub gene selection, validation, and analysis in order to screen potential biomarkers and therapeutic targets in both cardiomyopathies. In addition, we identified and verified ferroptosis-related genes in HCM and DCM, respectively, in order to uncover the underlying mechanisms between ferroptosis and cardiomyopathy. In the end, a total of 32 communal DEGs, 3 hub genes, and 4 ferroptosis-related genes that may provide new insights into the biological mechanisms and therapeutic directions of HCM and DCM were identified.

## Materials and Methods

### Data Source

The GEO (http://www.ncbi.nlm.nih.gov/geo) is a public database created by the US National Center for Biotechnology Information, which contains microarray and high-throughput sequencing datasets submitted by research institutes globally (19). HCM and DCM were used as keywords to search for gene-expression datasets with consideration of strict inclusion/exclusion criteria. The inclusion criteria were set as follows: [1] sporadic HCM or DCM, [2] datasets should consist of patients and healthy controls and should include the largest possible sample size, and [3] the test specimens in the datasets should be from human cardiac tissues. Exclusion criteria were as follows: Patients had participated in a clinical trial for drugs or other treatments. Finally, based on the inclusion/exclusion criteria, 6 microarray datasets were selected from GEO. In the DCM group, GSE36961 (106 patients with HCM and 39 controls), GSE32453 (8 patients with HCM and 5 controls), and GSE1145 (GPL570, 5 patients with HCM and 11 controls) were chosen from GEO, while GSE21610 (21 patients with DCM and 8 controls) (20), GSE79962 (9 patients with DCM and 11 controls) (21), and GSE3585 (7 patients with DCM and 5 controls) (22) were selected as the HCM datasets. GSE36961, GSE32453, GSE21610, and GSE79962 were used to identify DEGs, and GSE1145 and GSE3585 were used for hub gene and ferroptosis-related genes validation in subsequent analyses.

### Identification of DEGs

The GEO2R (https://www.ncbi.nlm.nih.gov/geo/geo2r/) is a network tool that works based on the Limma package and GEOquery (23). We used GEO2R to identify DEGs with the conditions of *P* < 0.05 and |logFC| > 0.5. Then, we constructed VEEN diagrams to detect and visualize the overlapping DEGs of the HCM and DCM datasets.

### Enrichment Analyses of Independent and Overlapping DEGs

We performed the Gene Ontology (GO) enrichment analysis to elucidate the biological characteristics of the independent DEGs using the database for annotation, visualization and integrated discovery (DAVID) database (https://david.ncifcrf.gov/) (24). Metascape (https://metascape.org) is used to conduct functional annotation analyses of overlapping DEGs (25) and further pathway enrichment of overlapping DEGs from 5 pathway databases (i.e., the Kyoto Encyclopedia of Genes and Genomes, Pathway Interaction Database, BioCyc, Reactome, and Panther) was implemented in the online platform KEGG orthology based annotation system (KOBAS) version 3.0. *P* < 0.05 was considered to be statistically significant (26).

### Protein–Protein Interaction Network Construction

Protein–protein interaction networks are used to uncover protein interactions and screen core protein genes. We constructed a PPI network of overlapping DEGs based on the STRING (http://string-db.org) version 11.0 database with the condition that the interaction combined score was > 0.4 points (27). Subsequently, Cytoscape (version 3.7.0) was used to visualize the molecular interaction networks of overlapping DEGs (28).

### Hub Gene Selection and Analyses

The hub genes in overlapping DEGs were screened using CytoHubba, a Cytoscape plugin, which was used to indicate the core protein genes of the PPI network (29). A total of 12 algorithms are available in CytoHubba, all of which are proved to be effective in identifying hub genes in this study. We randomly selected 5 of the 12 algorithms in CytoHubba and took the results of the intersection of these 5 algorithms to identify the hub genes. After that, a co-expression network of hub genes was constructed based on GeneMANIA (http://www.genemania.org/), which is a credible web tool for conducting gene list function analyses and unearthing internal associations (30). Finally, we predicted the subcellular localization of proteins encoded by the hub genes using the WoLF PSORT platform (31). Based on UniProt and the GO database, WoLF PSORT was able to predict the subcellular localization from the amino acid sequences of hub genes according to sorting signals, functional motifs, and amino acid composition.

### Validation of Hub Gene Expression

The hub gene expressions were verified in GSE1145 (GPL570 plus 5 patients with HCM and 11 controls) and GSE3585 (7 patients with DCM and 5 controls). A comparison between the 2 sets of data was performed with the *t*-test. *P* < 0.05 was considered to be statistically significant.

### Construction of the TF–mRNA–miRNA Regulatory Network

Mirwalk is a convenient database that mainly focuses on miRNA–target interactions (32). In this article, the miRNA of hub genes was predicted by the Mirwalk database with the strict condition that the predicted miRNA could be verified by other databases or experiments. The transcriptional Regulatory Relationships Unraveled by Sentence-based Text Mining (TRRUST) database, which contains the target genes corresponding to TFs and the regulatory relationships between TFs, was used to predict the TF that regulates the hub genes in this research (33). After the prediction of the TF–mRNA–microRNA (miRNA) relationship using TRRUST and Mirwalk, we used Cytoscape to visualize the regulatory network.

### Identification and Validation of Ferroptosis-Related DEGs in DCM and HCM

A total of 259 ferroptosis-related genes were obtained from the Ferroptosis Database (FerrDb; zhounan.org) (34), and we intersected these genes with DEGs of HCM to screen ferroptosis-related genes in HCM. To ensure the rigor and accuracy of this study, we verified these gene expressions in GSE1145. The comparison between the HCM and control sets of data was performed by the *t*-test, and *P* < 0.05 was considered to be statistically significant. Based on the validation results, we removed the disqualified genes and finally obtained the accurate ferroptosis-related genes in HCM. Using the same method, we acquired the ferroptosis-related genes of DCM, which were verified and proved to be significant in GSE3585.

## Results

### Identification of DEGs

**Figure 1:**
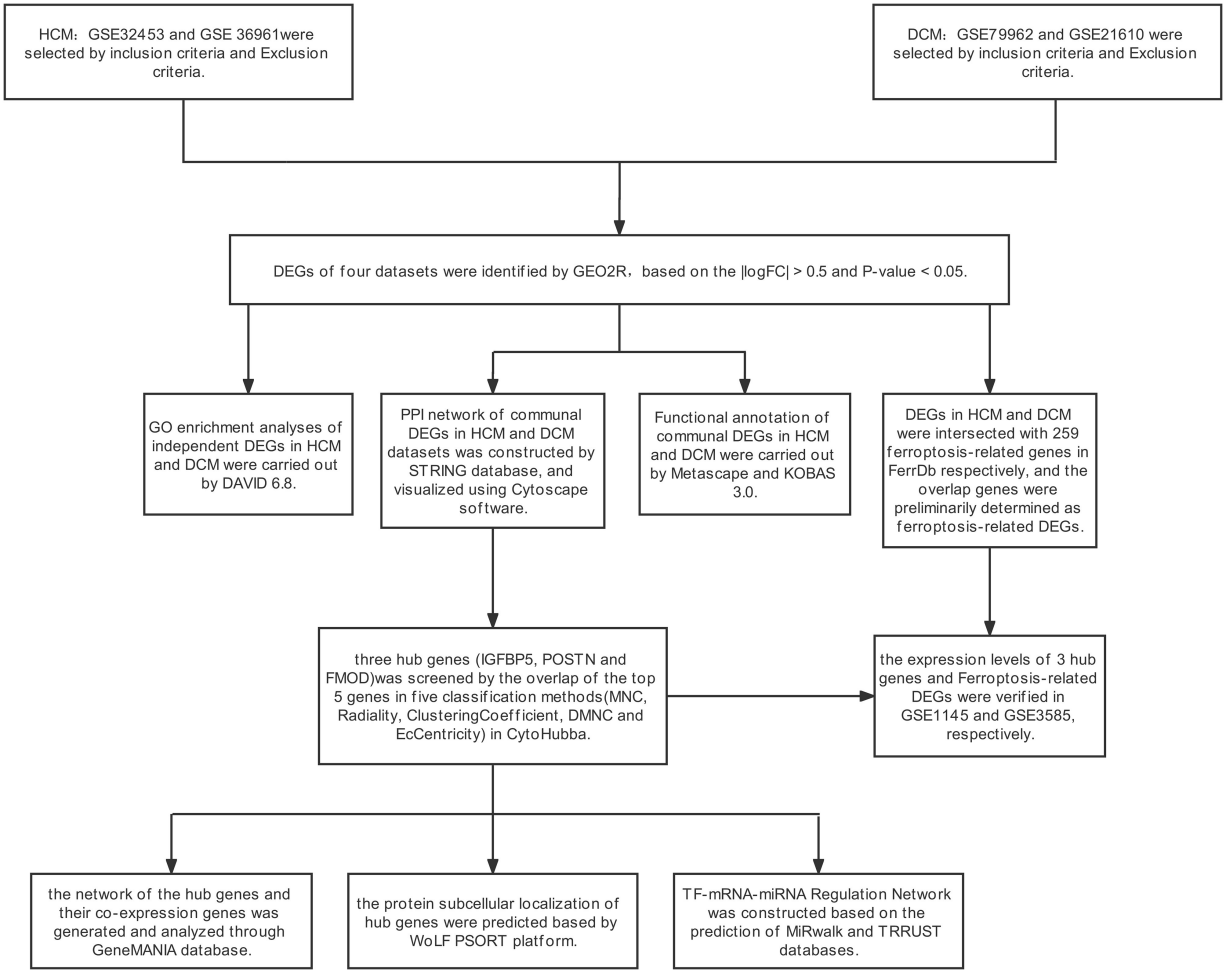
Research design flowchart.

**Figure 2:**
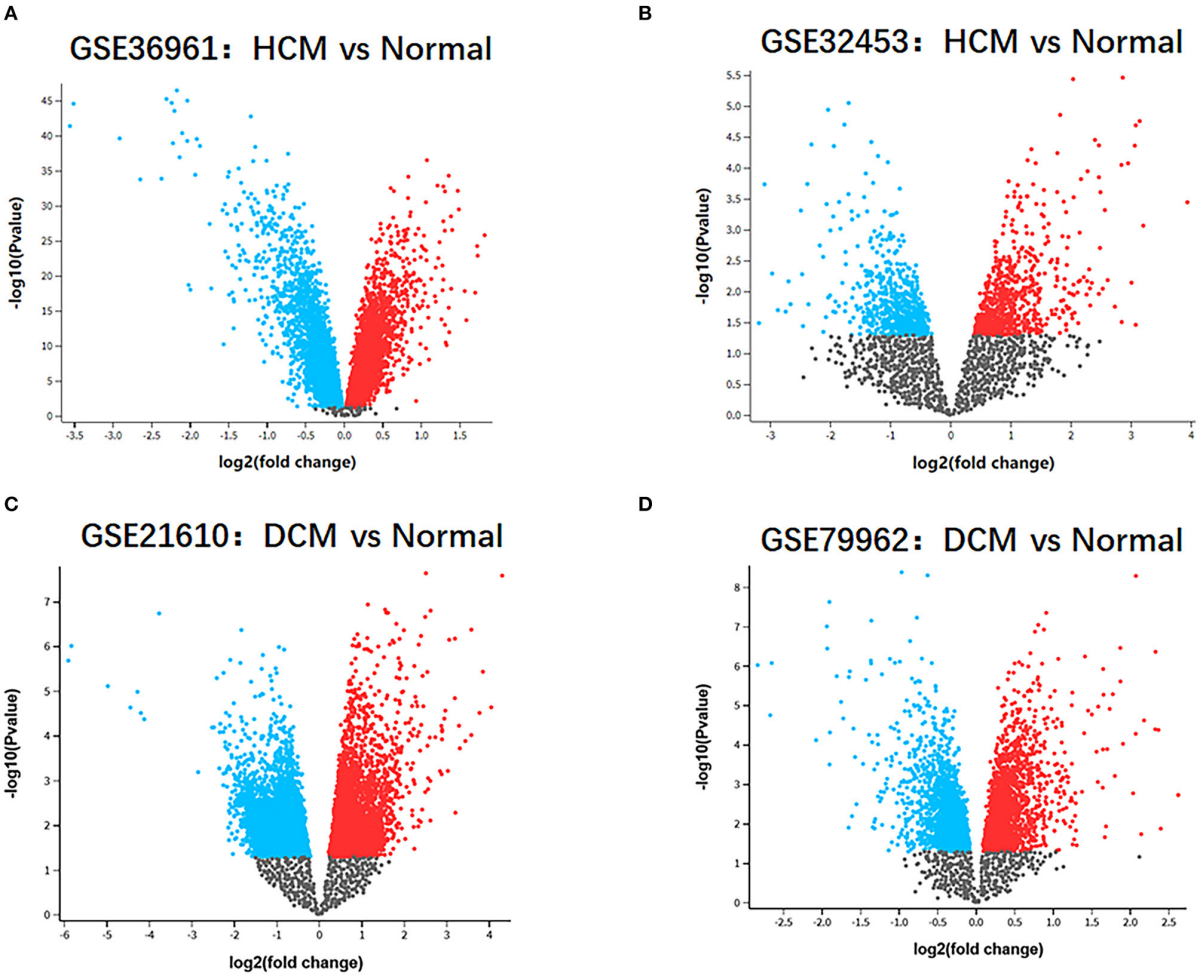
Identification of gene-expression profiles in the 4 datasets. **(A)** Volcano plot of GSE36961 microarray data. **(B)** Volcano plot of GSE32453 microarray data. **(C)** Volcano plot of GSE21610 microarray data. **(D)** Volcano plot of GSE79962 microarray data.

**Figure 3:**
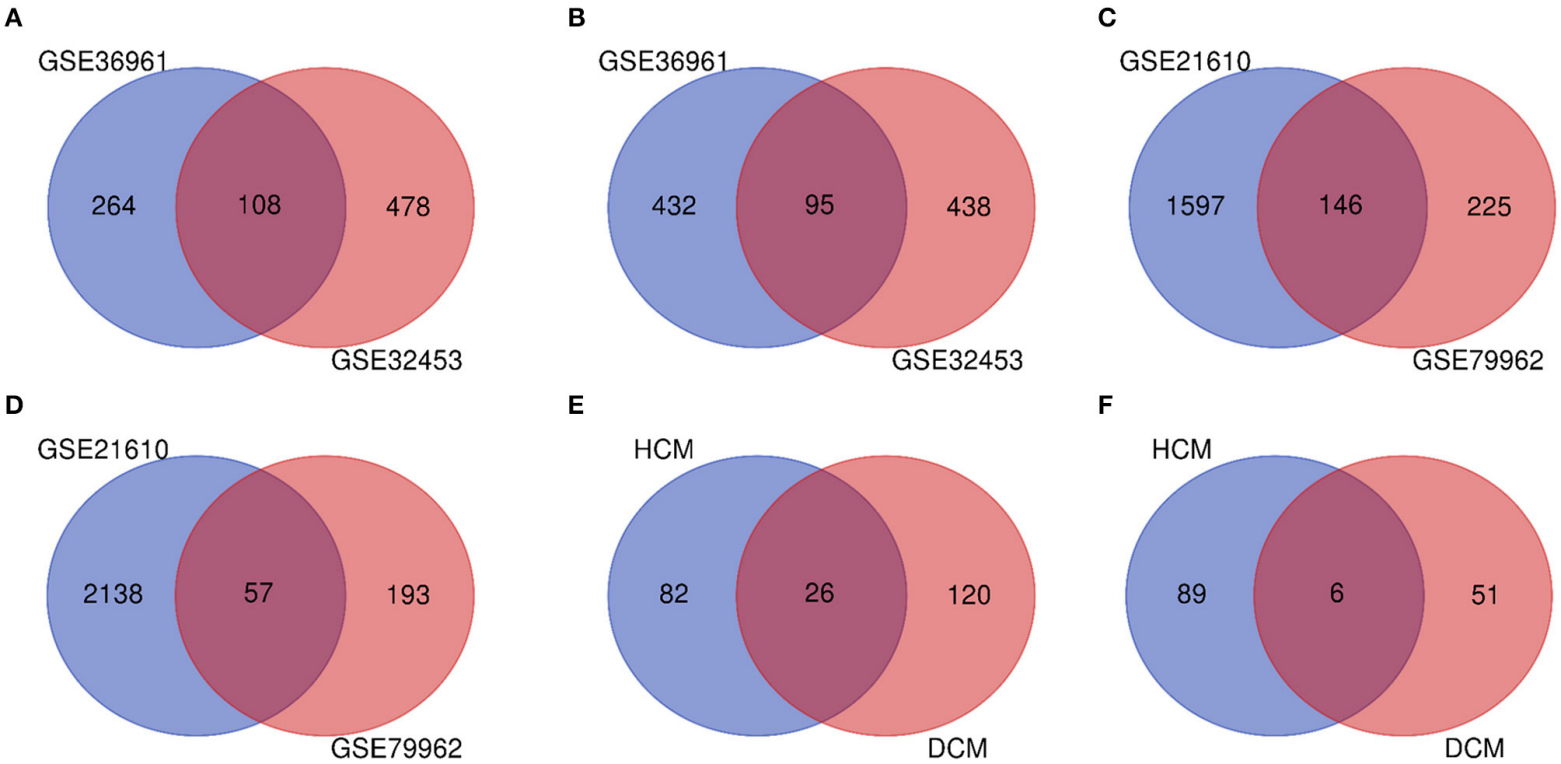
**(A)** Venn diagram of the 108 communal upregulated differentially expressed genes (DEGs) in HCM. **(B)** Venn diagram of the 95 communal downregulated DEGs in hypertrophic cardiomyopathy (HCM). **(C)** Venn diagram of the 146 communal upregulated DEGs in dilated cardiomyopathy (DCM). **(D)** Venn diagram of the 57 communal downregulated DEGs in DCM. **(E)** Venn diagram of the 26 communal upregulated DEGs shared between HCM and DCM. **(F)** Venn diagram of the 6 communal downregulated DEGs shared between HCM and DCM.

The research design flow chart is shown in Figure 1. After standardizing the microarray results, in the HCM group, the DEGs in GSE36961 (372 upregulated and 527 downregulated genes) (Figure 2A) and GSE32453 (586 upregulated and 533 downregulated genes) (Figure 2B) were screened, and DEGs1 (108 upregulated and 95 downregulated genes) were obtained by the intersection of DEGs in GSE32453 and GSE36961 (Figures 3A,B).

Meanwhile, in the DCM group, there were 3,938 DEGs in the GSE21610 dataset (1,743 upregulated and 2,195 downregulated genes) (Figure 2C) and 621 DEGs in the GSE79962 dataset (371 upregulated and 250 downregulated genes) (Figure 2D). We screened the DEGs of GSE79962 and GSE21610 to establish intersections and obtained DEGs2 (146 upregulated and 57 downregulated genes) (Figures 3C,D).

Differentially expressed genes 3 contained a total of 32 genes overlapping between DEGs1 and DEGs2, as shown in the Venn diagram, consisting of 26 upregulated genes and 6 downregulated genes (Figures 3E,F). Besides, 171 DEGs (DEGs4) and 171 DEGs (DEGs5) were identified independently from the DEGs3 in DEGs1 and DEGs2, respectively.

### Gene Ontology Enrichment Analyses of Independent DEGs in HCM and DCM

To uncover the biological roles of DEGs4 and DEGs5, the GO enrichment analysis was conducted (Table 1). The results were divided into 3 functional categories, specifically biological processes (BP), cell component (CC), and molecular function (MF). GO enrichment analysis of DEGs4 showed that the DEGs in the BP category were particularly involved in the positive regulation of ERK1 and ERK2 cascade (GO:0070374), chemotaxis (GO:0006935), actin filament organization (GO:0007015), and negative regulation of ERK1 and ERK2 cascade (GO:0070373). In terms of the CC category, DEGs4 were significantly involved in focal adhesion (GO:0005925), cytoplasm (GO:0005737), plasma membrane (GO:0005886), and clathrin-coated endocytic vesicle membrane (GO:0030669). As for the MF category, DEGs4 were mainly enriched in protein-binding (GO:0005515), protein homodimerization activity (GO:0042803), structural constituent of cytoskeleton (GO:0005200), and SH3/SH2 adaptor activity (GO:0005070). GO analysis results indicated that DEGs5 were mainly involved in cell adhesion (GO:0007155), positive regulation of cell-substrate adhesion (GO:0010811), positive regulation of cell division (GO:0051781), and positive regulation of cell proliferation (GO:0008284). In the CC category, the genes were particularly involved in the proteinaceous extracellular matrix (GO:0005578), extracellular space (GO:0005615), extracellular region (GO:0005576), and extracellular exosome (GO:0070062). Finally, the terms enriched under MF were collagen binding (GO:0005518), heparin binding (GO:0008201), integrin binding (GO:0005178), and calmodulin binding (GO:0005516).

**Table 1:** The gene ontology (GO) enrichment analysis of independent differentially expressed genes (DEGs) in hypertrophic cardiomyopathy (HCM) and dilated cardiomyopathy (DCM).

**DEGs**	**Ontology**	**ID**	**Description**	**Counts**	***P* value**
DEGs4	BP	GO:0070374	Positive regulation of ERK1 and ERK2 cascade	10	4.85E−05
		GO:0006935	Chemotaxis	8	1.74E−04
		GO:0007015	Actin filament organization	6	6.35E−04
		GO:0070373	Negative regulation of ERK1 and ERK2 cascade	5	0.002309719
	CC	GO:0005925	Focal adhesion	12	9.81E−04
		GO:0005737	Cytoplasm	66	0.002624132
		GO:0005886	Plasma membrane	54	0.00403869
		GO:0030669	Clathrin-coated endocytic vesicle membrane	4	0.006241558
	MF	GO:0005515	Protein-binding	110	2.44E−05
		GO:0042803	Protein homodimerization activity	17	0.001566078
		GO:0005200	Structural constituent of the cytoskeleton	6	0.003955574
		GO:0005070	SH3/SH2 adaptor activity	4	0.016052195
DEGs5	BP	GO:0007155	Cell adhesion	12	0.002514833
		GO:0010811	Positive regulation of cell-substrate adhesion	4	0.004515698
		GO:0051781	Positive regulation of cell division	4	0.008194139
		GO:0008284	Positive regulation of cell proliferation	11	0.008342239
	CC	GO:0005578	Proteinaceous extracellular matrix	16	9.53E−09
		GO:0005615	Extracellular space	33	8.94E−08
		GO:0005576	Extracellular region	35	5.31E−07
		GO:0070062	Extracellular exosome	47	4.96E−06
	MF	GO:0005518	Collagen-binding	7	1.05E−05
		GO:0008201	Heparin-binding	8	3.94E−04
		GO:0005178	Integrin-binding	6	0.001873305
		GO:0005516	Calmodulin-binding	7	0.005123577

### Functional Annotation of Communal DEGs in HCM and DCM

**Figure 4:**
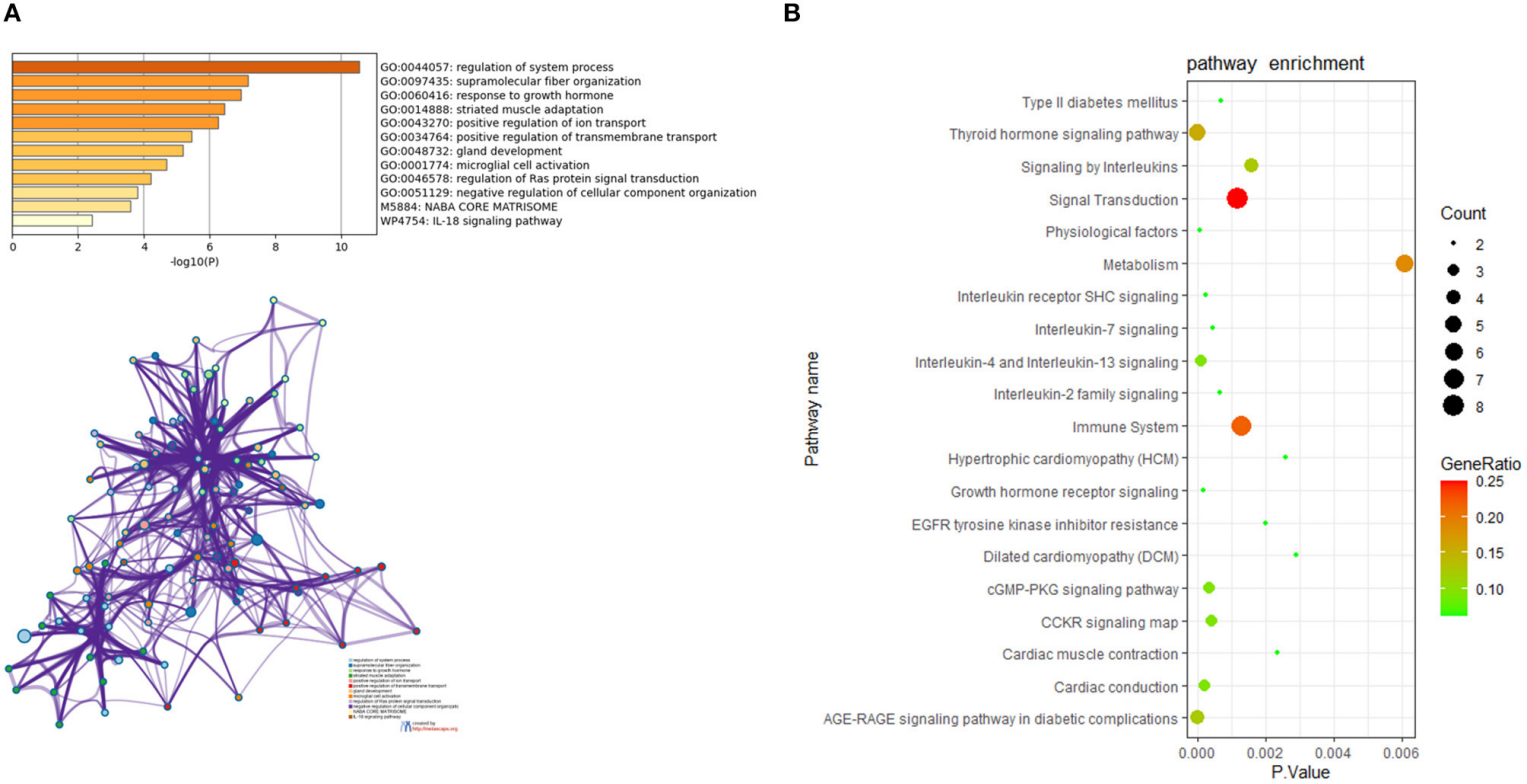
**(A)** The functional annotation analysis of the communal DEGs by Metascape. **(B)** The pathway analysis of the communal DEGs by KOBAS version 3.0.

For in-depth exploration and to reveal the biological characteristics of common DEGs in HCM and DCM, functional annotation analysis was performed with Metascape (Figure 4A), and further pathway enrichment analyses were conducted using KOBAS version 3.0 (Figure 4B). Functional annotation analysis using Metascape showed that DEGs5 were significantly involved in the regulation of system process (GO:0044057), supramolecular fiber organization (GO:0097435), and response to growth hormone (GO:0060416). Meanwhile, according to the further pathway enrichment analyses conducted with KOBAS version 3.0, DEGs5 were particularly enriched in signal transduction, immune system, signaling by interleukins, and growth hormone receptor signaling. These results revealed that growth hormone could play an important role in the communal mechanisms of HCM and DCM.

### Protein–Protein Interaction Network Construction and Hub Gene Selection and Analyses

**Figure 5:**
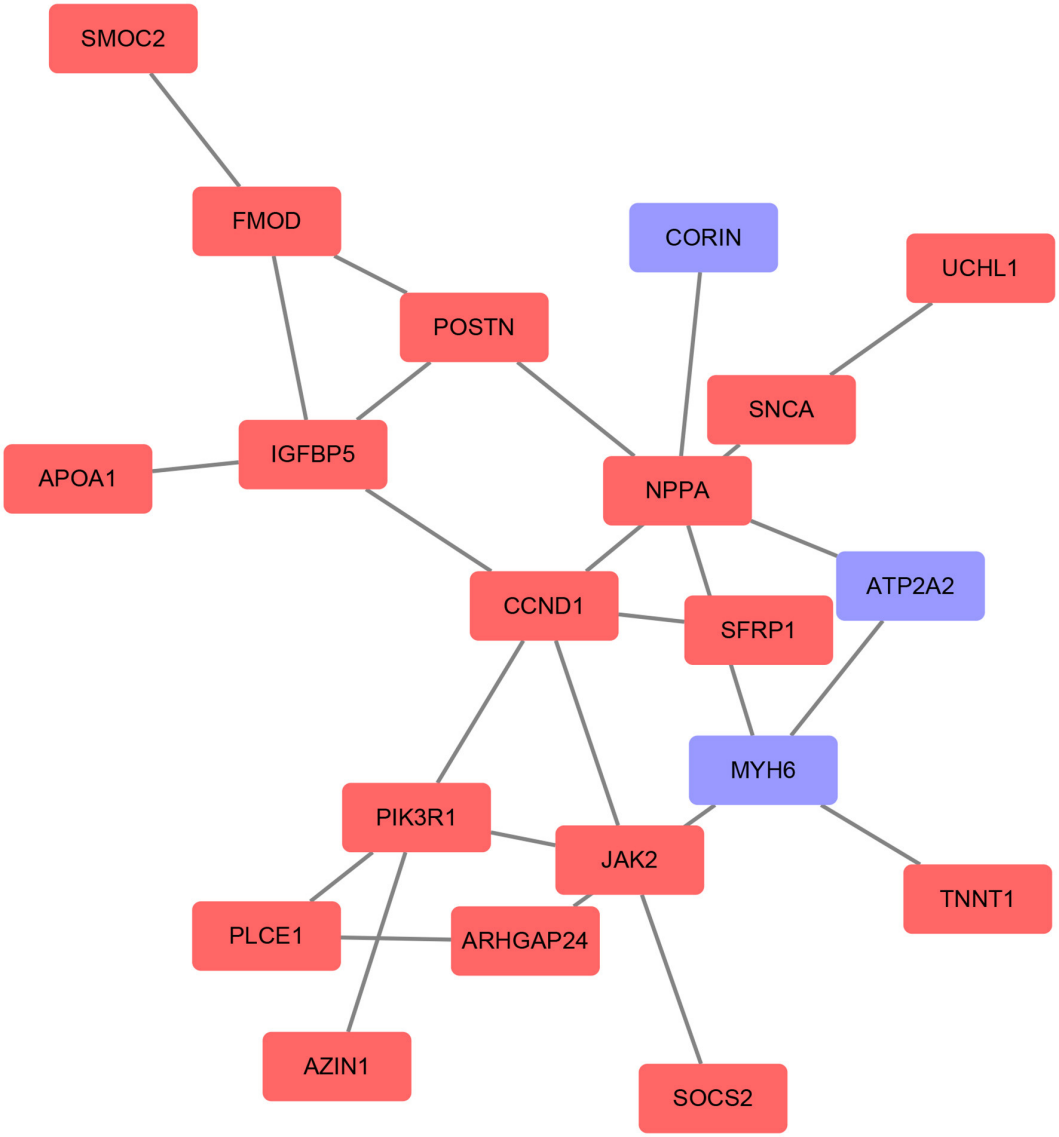
Protein–protein interaction (PPI) networks of the DEGs were constructed based on the STRING database and Cytoscape software. The red point represents upregulated genes and the blue point represents downregulated genes.

**Figure 6:**
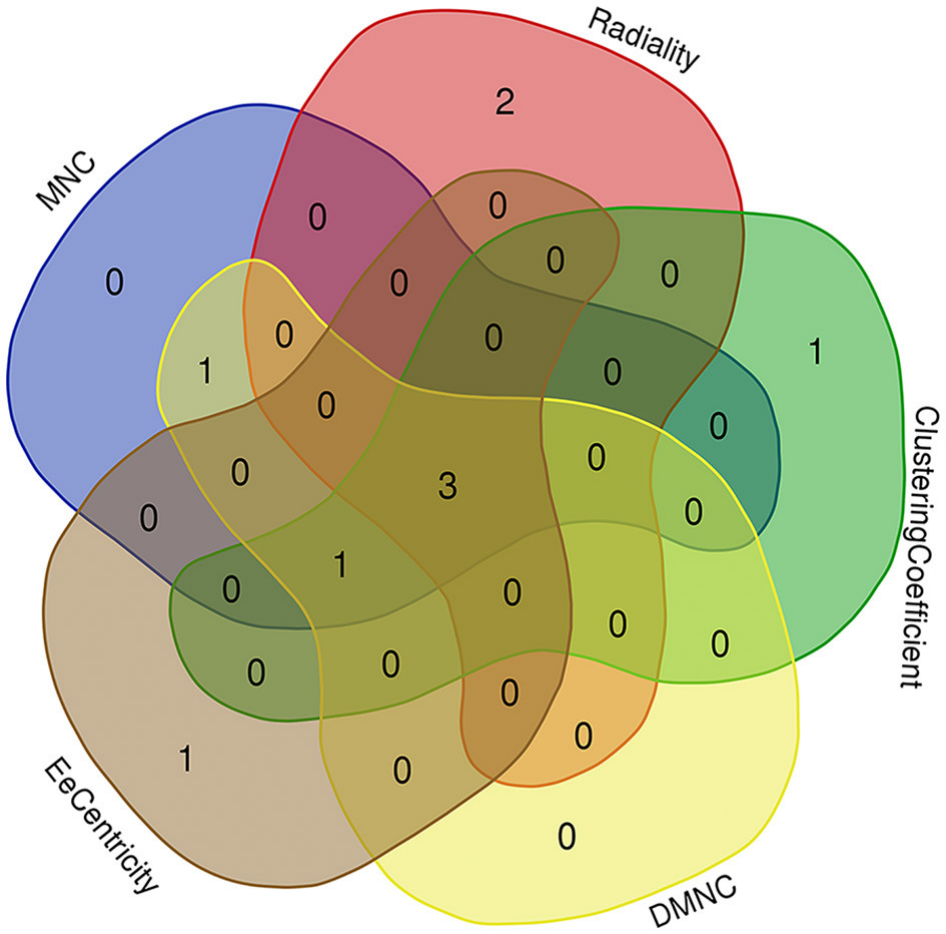
Three hub genes were screened by overlapping the top 5 genes found by the five algorithms of cytoHubba.

**Figure 7:**
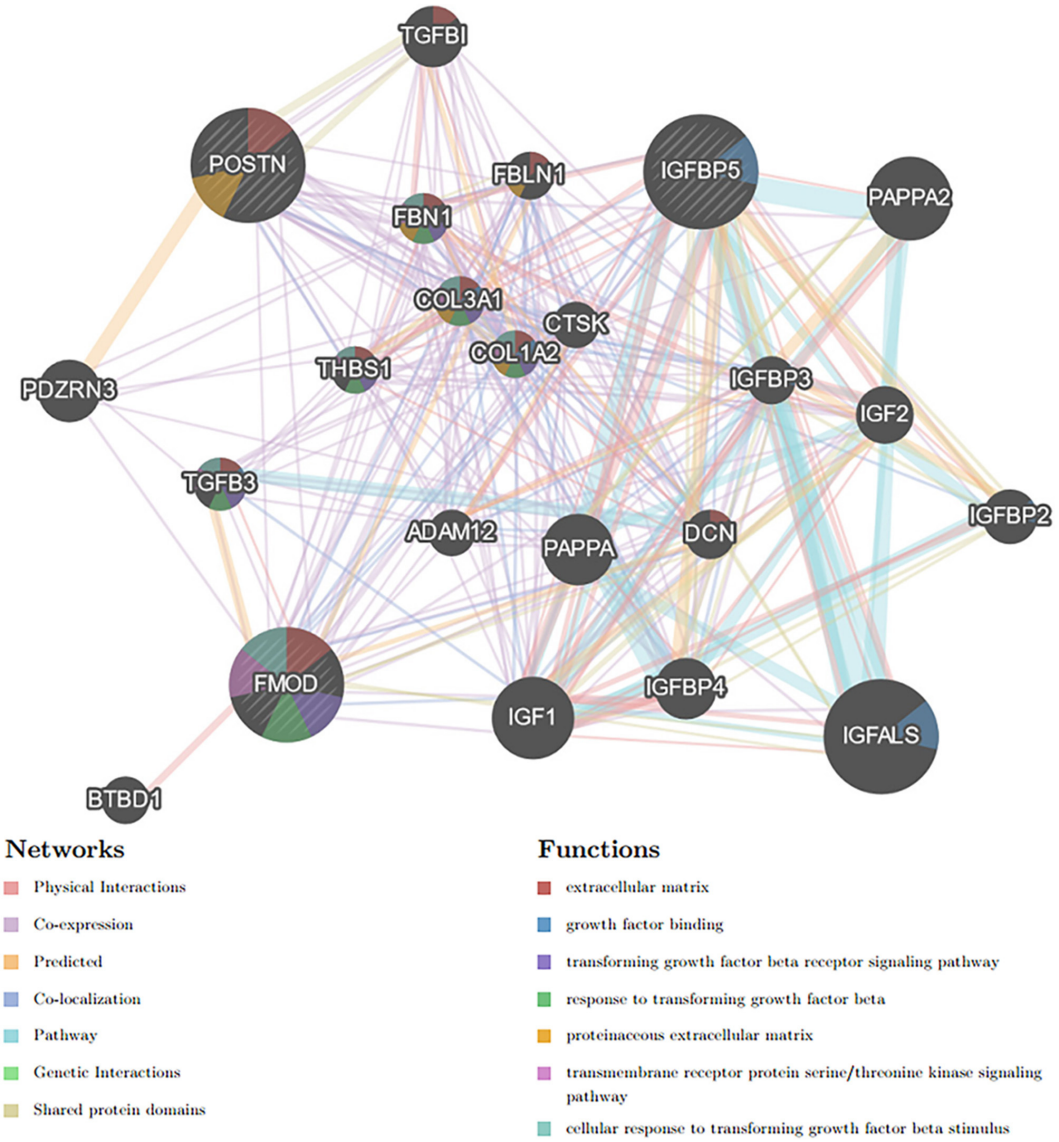
Hub genes and their co-expression genes were analyzed using GeneMANIA.

Based on the STRING database, the PPI network of DEGs5 with combined scores of >0.4 points was generated by Cytoscape, consisting of 20 nodes and 23 edges (Figure 5). In this article, 5 algorithms (MNC, Radiality, ClusteringCoefficient, DMNC, and EcCentricity) in CytoHubba were adopted to identify hub genes. The top 5 genes screened by these 5 algorithms are listed in Table 2. Subsequently, we overlapped the top 5 genes found by the 5 algorithms to determine the central genes (Figure 6). Three upregulated genes [insulin-like growth factor-binding protein-5 (*IGFBP5*), periostin (*POSTN*), and fibromodulin (*FMOD*)] were screened as hub genes. In order to better uncover the biological characteristics of these hub genes, we constructed and analyzed the network of the hub genes and their co-expression genes based on the GeneMANIA platform (Figure 7). Three hub genes showed a complex PPI network with physical interactions of 67.64%, co-expression of 13.50%, co-localization of 6.17%, prediction of 6.35%, pathway of 4.35%, genetic interactions of 1.40%, and shared protein domains of 0.59%. As expected, the biological function of the hub genes re-emphasizes the importance of growth hormone and extracellular matrix in HCM and DCM, and these results may provide clues about therapeutic directions in cardiomyopathies. In addition, the subcellular localization of proteins encoded by the 3 hub genes suggested that *FMOD* and *IGFBP5* exist in extracellular areas, whereas *POSTN* is located in the endoplasmic reticulum.

**Table 2:** The top 5 hub genes as ranked in cytoHubba.

**MNC**	**Radiality**	**ClusteringCoefficient**	**DMNC**	**EcCentricity**
JAK2	FMOD	JAK2	JAK2	JAK2
MYH6	PIK3R1	ATP2A2	MYH6	FMOD
FMOD	POSTN	FMOD	FMOD	SMOC2
POSTN	CCND1	POSTN	POSTN	POSTN
IGFBP5	IGFBP5	IGFBP5	IGFBP5	IGFBP5

### Validation of Hub Gene Expression

**Figure 8:**
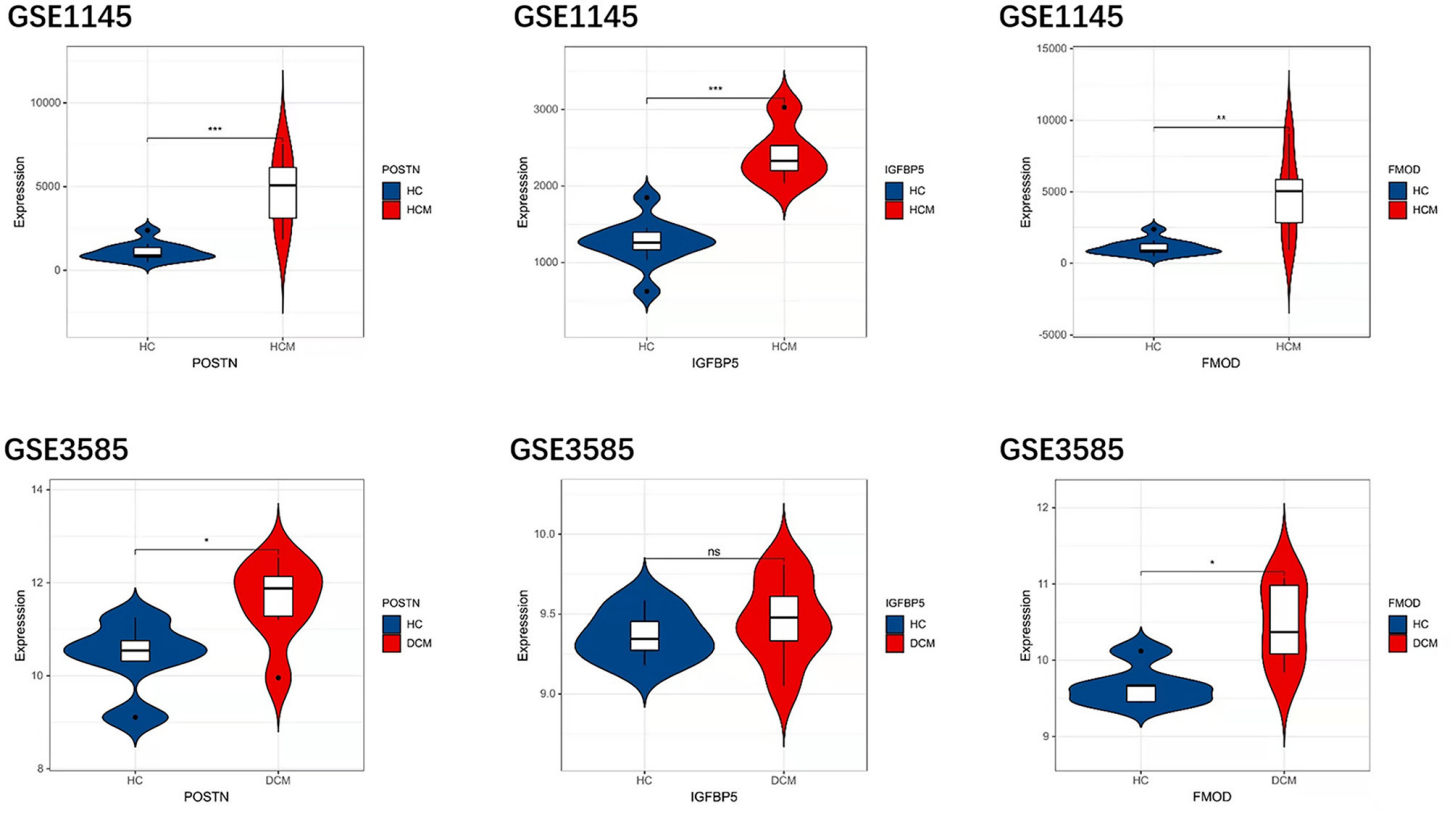
The expression levels of hub genes in GSE1145 and GSE3585. The comparison between the two sets of data used the mean *t*-test. *P* < 0.05 was considered to be statistically significant. HC, healthy control. *P < 0.05; **P < 0.01; ***P < 0.001.

In order to prove the reliability and accuracy of the bioinformatics analysis results, GSE1145 and GSE3585 were used to verify the expression of hub genes in HCM and DCM samples by independence testing analysis respectively (Figure 8). The results showed that the expression levels of all the 3 hub genes in HCM heart tissue were significantly higher than in normal heart tissue. In DCM heart tissue, *FMOD* and *POSTN* were significantly upregulated compared to in normal heart tissue, However, IGFBP5 were not seemed to change significantly in GSE3585.

### TF–mRNA–miRNA Regulation Network Construction

**Figure 9:**
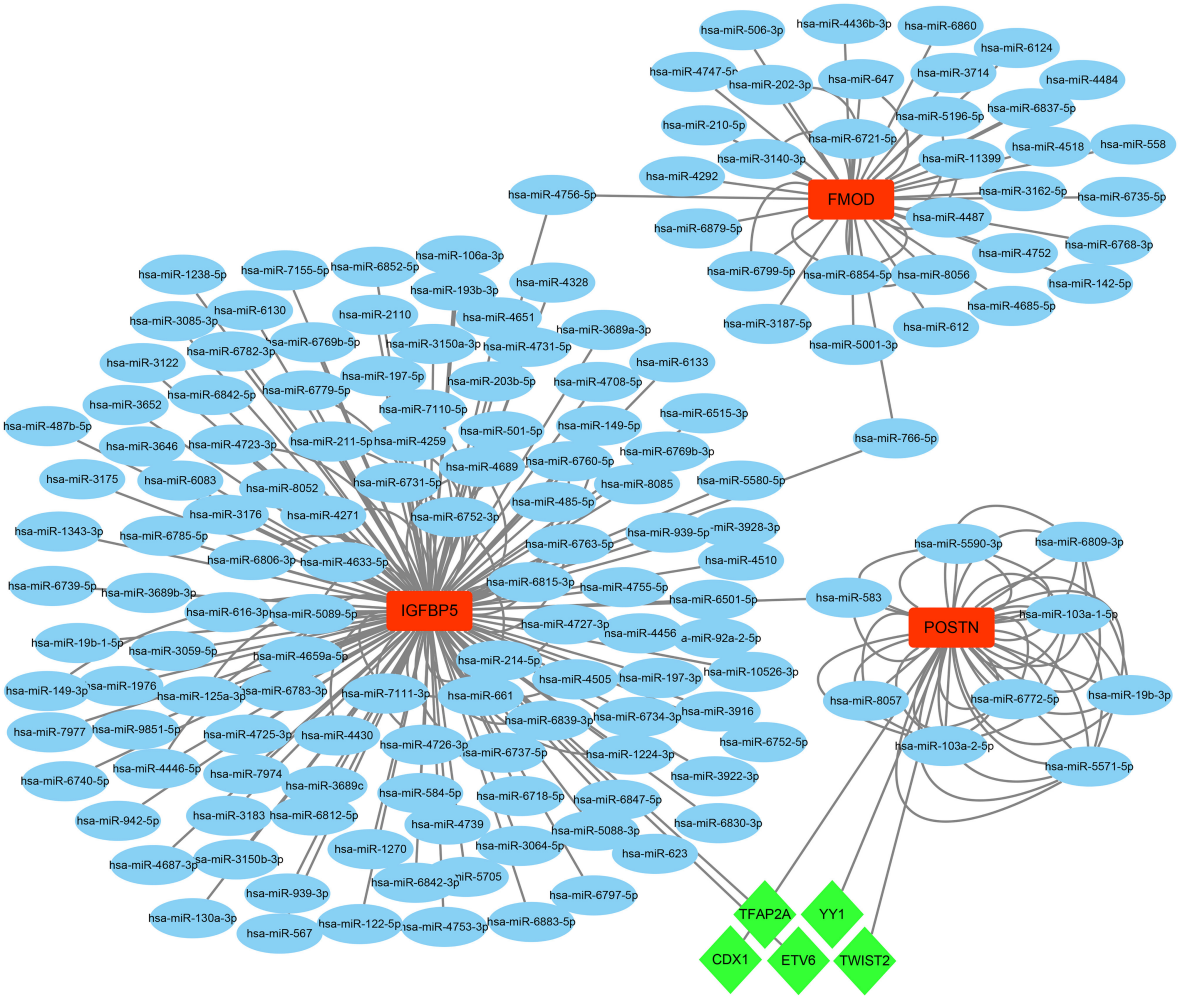
The TF–mRNA–miRNA regulation network of hub genes was constructed using the MiRwalk and TRRUST databases.

On the grounds of the MiRwalk database predictions of the 3 hub genes, a total of 236 miRNAs were obtained with the condition that their prediction could be verified by experiments or other databases. We screened 5 TFs [YY1 transcription factor (YY1), transcription factor AP-2 alpha (TFAP2A), caudal type homeobox 1 (CDX1), ETS variant transcription factor 6 (ETV6), and twist family bHLH transcription factor 2 (TWIST2)] that could regulate the expression of the 3 hub genes based on the TRRUST database. Subsequently, a regulatory network of hub genes and their predicted miRNAs and TFs were generated by the Cytoscape software (Figure 9).

### Identification and Validation of Ferroptosis-Related DEGs in DCM and HCM

**Figure 10:**
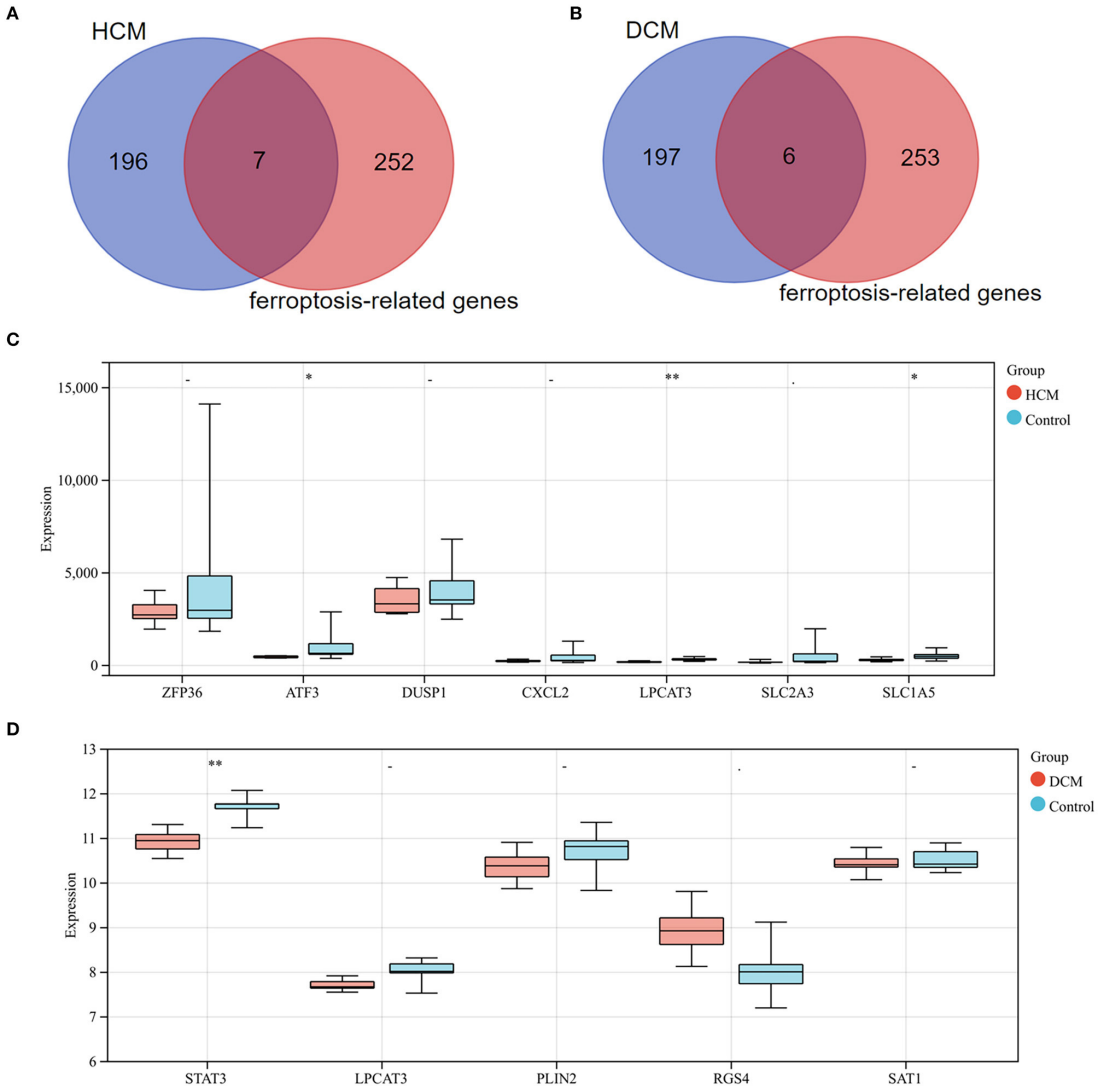
**(A)** Venn diagram of preliminary ferroptosis-related DEGs in HCM. **(B)** Venn diagram of preliminary ferroptosis-related DEGs in DCM. **(C)** The expression levels of preliminary ferroptosis-related DEGs of HCM in GSE1145. **(D)** The expression levels of preliminary ferroptosis-related DEGs of DCM in GSE3585. *P < 0.05; **P < 0.01.

Differentially expressed genes1 were intersected with 259 ferroptosis-related genes and 7 genes were screened as a result. All the 7 genes were downregulated DEGs, including *ZFP36* ring finger protein (*ZFP36*), activating transcription factor 3 (*ATF3*), dual specificity phosphatase 1 (*DUSP1*), C-X-C motif chemokine ligand 2 (*CXCL2*), lysophosphatidylcholine acyltransferase 3 (*LPCAT3*), *SLC2A3*, and solute carrier family 1 member 5 (*SLC1A5*). Subsequently, these genes' expression was verified in GSE1145, and *ATF3, LPCAT3*, and *SLC1A5* were determined to be significant ferroptosis-related genes in HCM. In the same way, we took the intersection of DEGs2 and 259 ferroptosis-related genes and obtained 2 upregulated genes [regulator of G protein signaling 4 (*RGS4*) and arrestin domain containing 3 (*ARRDC3*)] and 4 downregulated genes [*STAT3, LPCAT3*, perilipin 2 (*PLIN2*), and spermidine/spermine N1-acetyltransferase 1 (*SAT1*)]. Subsequently, *STAT3* was determined to be a ferroptosis-related gene in DCM based on validation in GSE3585 (Figure 10).

## Discussion

In this article, we screened 26 upregulated and 6 downregulated overlapping DEGs in HCM and DCM. Functional annotation analysis revealed that DEGs are mainly involved in the immune system and growth hormone. After that, three hub genes (*IGFBP5, POSTN*, and *POSTN*) were determined in the PPI network. Subsequently, we constructed and analyzed a network of hub genes and their co-expression genes. The biological functions of the hub genes showed that growth hormone and extracellular matrix play important roles in the pathogenesis of DCM and HCM. Finally, we verified hub gene expression and constructed a TF–mRNA–miRNA regulation network.

Notably, both the GO and pathway analysis of communal DEGs and hub genes highlighted the important position of the growth hormone. In the cardiovascular system, GH plays a role as a modulator with certain effects in regulating cardiac growth and metabolism (35, 36). In recent years, numerous studies have focused on the association between the GH system and HF, especially HF caused by cardiomyopathy. Arcopinto et al. (37) reported that patients with chronic HF with GH deficiency show larger LV volumes, worse cardiac function, and increased mortality compared to patients with GH sufficient. Despite that GH treatment for HF was proven to be beneficial in various animal models, there remain controversies about the efficacy when translated into the clinical field (38). Cittadini et al. (39) showed that GH therapy in patients with HF could improve LV volume and ejection fraction, but Isgaard et al. (40) reported that no beneficial effects on cardiac function or structure could be detected in patients with HF receiving GH treatment. GH resistance was used to explain the variations in GH treatment efficacy, yet it is still far from elucidating the exact mechanism (41). Meanwhile, GH treatment for patients with DCM could increase the LV wall thickness and reduce the chamber size (42), but there is still a lack of relevant experiments and evidence in the area of treatment for HCM. In conclusion, consistent with our analysis, previous studies and evidence have preliminarily suggested that GH could be a potential clinical biomarker or therapeutic direction for cardiomyopathy and HF, but more studies are needed to confirm these suggestions urgently.

Periostin encodes a secreted extracellular matrix protein that functions in tissue development and regeneration, and the encoded protein binds to integrins to support the adhesion and migration of epithelial cells (43). Recently, numerous studies have shown that *POSTN* plays an important role in cardiovascular disease. Katsuragi et al. (44) found that a high level of *POSTN* expression causes a decrease in cardiac myocytes and an increase in collagen deposition, which results in cardiac insufficiency. LV function could be effectively improved and the survival rate can be increased by inhibiting *POSTN* gene expression in a rat model. In addition, previous studies have also speculated and proved that POSTN may be a mediator of cardiac remodeling (45, 46). With the deepening of research, we need to pay more attention to the effects of *POSTN* in cardiomyopathy. There is significant evidence that the expression of *POSTN* is both increased in HCM and DCM and has different effects in both aspects and degrees. Norum et al. (46) found that mRNA and protein levels of POSTN in the plasma and heart tissues of patients with DCM are higher than in those of healthy controls, and the poorer the heart function was, the higher the levels were. In addition, some studies suggest that the level of *POSTN* could affect the efficacy of some treatment methods in patients with DCM, such as cardiac resynchronization therapy (47). Meanwhile, *POSTN* expression was verified to be increased in an HCM mouse model (48), and other studies suggest that *POSTN* may promote fibrosis and collagen deposition in patients with HCM through transforming growth factor (TGF)-β signaling, which is known to be activated in various HCM models (49, 50). As above, it is undeniable that *POSTN* could be a potential biomarker or target for cardiomyopathy.

Insulin-like growth factor-binding protein-5 is a member of family IGF-binding proteins (IGFBP) family. In the past 30 years, studies of IGFBP5 have mainly focused on the regulatory mechanisms of kidney, bone, and mammary gland physiological processes and the regulation of specific tumor proliferation (51). With deepening research, IGFBP5 has been proven to play an important role in the biological mechanism of fibrosis (52). Song et al. (53) found that IGFBP5 mediates high glucose-induced profibrotic effects in cardiac fibroblasts and Yasuoka et al. (54) reported that injection of an adenovirus expressing human IGFBP5 into mice induced skin fibrosis. Furthermore, IGFBP5 can bind insulin-like growth factor 1 (IGF-1) and block the activation of IGF-1 signaling (55). IGF-1 is considered to be a protective factor for the heart, especially in cardiomyopathy. The expression of IGF-1 was increased in both patients with HCM and DCM, and the degree of increase was related to the extent of both myocardial injury and recovery (56, 57). IGF-1 may act in concert with stromal cell-derived factors to protect and improve cardiac structure and function by reducing apoptosis and promoting repair (57, 58). Meanwhile, some studies have reported that IGF-1 treatment could effectively improve cardiac function and the survival rate in animal models of cardiomyopathy (59, 60). To sum up, we speculate that *IGFBP5* could be a potential therapeutic target.

Fibromodulin belongs to the family of small interstitial proteoglycans. Its encoded protein (FOMD) may play a role in the assembly of extracellular matrix and regulate TGF-β activities by sequestering transforming growth factor-β (TGF-β) into the extracellular matrix (61). As is known, cardiac fibroblasts play an important role in cardiac fibrosis (62). Cardiac fibroblasts were proven to express *FOMD*, and the expression level was increased under pro-inflammatory stimuli. High expression of *FOMD* inhibits and reduces the migration of cardiac fibroblasts so as to alleviate cardiac fibrosis (63, 64). In the heart tissue of patients with HF and mice, the expression of *FMOD* was upregulated 3–10 times. Andenaes et al. (63) found that the LVs of *FMOD*-knockout mice developed mildly exacerbated hypertrophic remodeling compared to controls under aortic banding, and *FMOD* has anti-fibrotic functions in cultured cardiac fibroblasts. Therefore, *FOMD* could be a potential marker and offer new therapeutic methods.

In order to prove the reliability and accuracy of our bioinformatics analysis results, hub gene expression was verified in other gene-expression datasets, and the results confirm the validity of the analysis in this research. Since the changes of *IGFBP5* in the DCM group of GSE3585 were not significant, we retain some doubts about the critical role of *IGFBP5* in DCM. In addition, we used the MiRwalk and TRRUST databases to predict a total of 236 miRNAs and 5 TFs of hub genes, which might show the potential mutual regulation between genes, allowing us to better understand the relationship and the potential regulation between hub genes.

In this study, we also identified and verified ferroptosis-related DEGs in HCM and DCM, respectively, in order to uncover the underlying mechanisms, which could provide a novel direction for exploration. *STAT3*, a transcription factor signal transducer and activator of transcription 3, has been linked to many cardiac protective mechanisms. Recently, more and more studies have identified that the cardiac myocyte *STAT3* plays an important role in maintaining metabolic homeostasis (65). Researchers have proven that the protective effects of *STAT3* are the key to inducing anti-inflammatory and survival gene expression (66). However, the exact molecular mechanism of *STAT3* in cardiac protection remains unclear. Ferroptosis, an iron-dependent process, is a newly revealed type of cellular programmatic death that is different from apoptosis, autophagy, and necrosis. Meanwhile, *STAT3* is known to be an inhibitor of ferroptosis due to suppressing the expression of ACSL4, which is an essential enzyme required for ferroptosis (67, 68). Based on our results, we boldly hypothesize that ferroptosis plays a significant role in DCM and may be regulated by *STAT3*, which provides a new direction for DCM treatment in the future.

Next, we speculated whether ferroptosis also works in HCM, so we analyzed the association between HCM and ferroptosis, same as we did with DCM. We found that 3 genes make sense, including *ATF3, LPCAT3*, and *SLC1A5*. *ATF3*, a member of the basic leucine zipper superfamily of transcription factors, was identified as an adaptive-response gene under stress conditions (69). In the cardiovascular system, it is significant in modulating cardiac remodeling, as mice with *ATF3* deficiencies showed cardiac hypertrophy, dysfunction, and fibrosis under overload pressure (70). What's more, Wang et al. (71) found that *ATF3* could suppress system Xc^−^ and predispose cells to ferroptosis by repressing SLC7A11 expression. Orchestrally, *LPCAT3* and *SLC1A5* are reported to participate in the ferroptosis pathway (67, 72) and facilitate ferroptotic cell death. However, the exact nature of the mechanism of these ferroptosis-related genes is far from clear, especially in cardiomyopathy, and there may still be some potential mechanisms that need to be elucidated to explain this phenomenon. In contrast to that in DCM, the role of ferroptosis in HCM seems more complex, and how ferroptosis influences HCM is still unknown. Anyway, we herein suggest the potential role of ferroptosis and the conceivable target genes in DCM and HCM, which could serve as a cardioprotective strategy for cardiomyopathy prevention. However, gaining more detailed and accurate knowledge of the mechanism behind the process requires further research.

In this article, we highlighted the potential role of hub genes and screened the ferroptosis-related genes in two cardiomyopathies. However, we acknowledge that the research has some limitations. First, despite the analysis involving a slightly large sample size and our success in verifying the expression hub genes and ferroptosis-related genes in other gene-expression datasets, this article is a retrospective study that still requires external verification. Moreover, the biological functions of these genes need to be further verified in an *in vitro* model. The above study will be the focus of our future work.

## Conclusion

In summary, the revealed independent DEGs provide us with new insight into the distinct mechanisms of HCM and DCM, and the common DEGs screened in this study help to uncover a potential common mechanism of HCM and DCM. In addition, ferroptosis-related genes could provide us with a novel direction of exploration in HCM and DCM, respectively, and 3 hub genes (*POSTN, IGFBP5*, and *FMOD*) could be potential biomarkers or therapeutic targets in cardiomyopathies.

## Data Availability Statement

The datasets presented in this study can be found in online repositories. The names of the repository/repositories and accession number(s) can be found in the article/Supplementary Material.

## Author Contributions

All authors listed have made a substantial, direct, and intellectual contribution to the work and approved it for publication.

## Funding

This study was supported by the Clinical Trial Ability Improvement Project of the Cardiovascular Professional Group of The First Affiliated Hospital of Soochow University (No. 201900180019).

## Conflict of Interest

The authors declare that the research was conducted in the absence of any commercial or financial relationships that could be construed as a potential conflict of interest.

## Publisher's Note

All claims expressed in this article are solely those of the authors and do not necessarily represent those of their affiliated organizations, or those of the publisher, the editors and the reviewers. Any product that may be evaluated in this article, or claim that may be made by its manufacturer, is not guaranteed or endorsed by the publisher.
